# Can Financial Institutional Deepening and Renewable Energy Consumption Lower CO_2_ Emissions in G-10 Countries: Fresh Evidence from Advanced Methodologies

**DOI:** 10.3390/ijerph19095544

**Published:** 2022-05-03

**Authors:** Usman Mehmood, Salman Tariq, Zia ul Haq, Ephraim Bonah Agyekum, Salah Kamel, Mohamed Elnaggar, Hasan Nawaz, Ammar Hameed, Shafqat Ali

**Affiliations:** 1 Remote Sensing, GIS and Climatic Research Lab, National Center of GIS and Space Applications, Centre for Remote Sensing, University of the Punjab, New-Campus, Lahore 54590, Pakistan; usmanmehmood.umt@gmail.com (U.M.); zia.spsc@pu.edu.pk (Z.u.H.); hasan92nawaz@gmail.com (H.N.); ammarmian56@gmail.com (A.H.); shafqat.rsgcrl@pu.edu.pk (S.A.); 2 Department of Political Science, University of Management and Technology, Lahore 54770, Pakistan; 3 Remote Sensing, GIS and Climatic Research Lab, National Center of GIS and Space Applications, Department of Space Science, University of the Punjab, New-Campus, Lahore 54590, Pakistan; salman.spsc@pu.edu.pk; 4 Department of Nuclear and Renewable Energy, Ural Federal University Named after the First President of Russia Boris Yeltsin, 620002 Ekaterinburg, Russia; agyekumephraim@yahoo.com; 5 Department of Electrical Engineering, Faculty of Engineering, Aswan University, Aswan 81542, Egypt; skamel@aswu.edu.eg; 6 Department of Electrical Engineering, College of Engineering, Prince Sattam Bin Abdulaziz University, Al-Kharj 16273, Saudi Arabia; 7 Department of Electrical Power and Machines Engineering, Faculty of Engineering, Helwan University, Helwan 11795, Egypt

**Keywords:** CO_2_ emissions, G-10 countries, GDP, renewable energy consumption, financial institutional deepening

## Abstract

To tackle the challenges associated with global warming and climate change, several countries set their targets to lower carbon emissions in accordance with COP21 (Paris Conference). Even though studies highlighted the different aspects that contribute to environmental degradation, there still exists the scarcity of adequate research that emphasizes the environmental implications of financial institutional deepening, renewable energy consumption (REC), and technology innovations. Therefore, this study investigated the significance of financial institutional deepening, REC, gross domestic product (GDP), imports, exports, and technology innovations to achieve sustainability in G-10 countries, namely The Netherlands, Germany, France, Switzerland, United Kingdom, Sweden, Japan, Belgium, Canada, and Italy from 1990 to 2020. The results obtained from cross-sectionally augmented autoregressive distributed lag (CS-ARDL) and the dynamic common correlated effects mean group (DCCEMG) models reveal that financial institutional deepening and imports positively impact CO_2_ emissions (CO_2_e) both in the long and short run. A 1% increase in financial institutional deepening and import will increase CO_2_e by 0.5403% and 0.2942% in the short run and 0.2980% and 0.1479% in the long run levels, respectively. Contrary to this, REC, GDP, exports, and technology innovations improve environmental quality in these countries. The Dumitrescu & Hurlin causality test shows bidirectional causality between imports and CO_2_e, GDP and CO_2_e, exports and CO_2_e, and financial institutional deepening and CO_2_e, compared to unidirectional causality from technology innovations to CO_2_e and from REC to CO_2_e. Apart from this, the outcomes suggest that policymakers in G-10 countries have to consider their financial markets and firms to revise their current environmental policies.

## 1. Introduction

Over the last few decades, the unsustainable effect of environmental degradation has become a serious issue that significantly influences human wellbeing, ecosystems and the nature of the atmosphere [1,2,3,4,5]. Rapid urbanization and industrialization resolves problems of human settlement and poverty at the cost of destruction of the environment [6,7,8]. The increasing challenges of extreme temperatures, changes in weather patterns, snow and glaciers melting, rising sea levels and climate change [6,9,10,11,12,13,14], and human health impacts [15,16,17,18,19,20] have become the focus of social and energy experts, econometrists, and environmentalists. 

Global warming and climate change has been linked with increasing greenhouse gases (GHG) and carbon dioxide emissions (CO_2_e). Therefore, several protocols has been signed among nations, such as [21,22,23] to keep global temperatures below 2 °C, preferably 1.5 °C. These agreements impose certain limitations to lower GHG and CO_2_e. G-10 countries i.e., The Netherlands, Germany, France, Switzerland, United Kingdom, Sweden, Japan, Belgium, Canada, and Italy that have major contribution towards CO_2_e have committed to achieve net zero carbon emissions. Carbon neutrality is a situation based on PAS 2060 of the British Standard Institution, that has net zero total annual CO_2_e from all anthropogenic sources [24]. Several countries have set their objectives to achieve carbon neutrality by 2050 through reduction of GHG emissions by 80 to 100% [25]. For example, the Swedish Government introduced a Climate Act Framework to reach net zero carbon emissions by 2045, five years earlier than the rest of G-10 countries. The Canadian government introduced Zero Carbon Buildings Framework to lower GHG emissions from commercial, institutional, and residential sectors. The Canada Green Building Council aims to reduce GHG emissions up to 30% by 2030. In COP26, the United States of America plans to employ innovative technologies and encourage use of sustainable fuels (hydrogen and biofuels) and renewable energy sources to hit the net zero target. They are also working on the protection of forests as natural carbon sinks [26].

By the end of 2018, the global energy consumption (EC) increased by ~2.3%, in which ~80% of total world energy was produced from fossil fuels [2]. According to [27], all nations should adopt an alternative to fossil fuels, such as renewable energy resources for energy production, to attain carbon neutrality. They also recommend deploying energy- efficient technologies in different economic and industrial sectors to reduce carbon emissions. The G-10 countries are the largest importers and exporters of carbon commodities. Also, they are making huge investments in technology innovations and clean, green energy projects to lower carbon emissions. Moreover, in G-10 countries, the carbon tax policy discourages the use fossil fuels and import of high CO_2_e goods, thus urging organizations to transition to clean energy resources. Thus, assisting G-10 economies to achieve net zero carbon emissions targets by or before 2050 is the goal. 

International commerce is also crucial to gross domestic product (GDP) development in G-10 countries. Their contribution gives us a realistic view of CO_2_e and is also essential for attaining carbon neutrality. According to [28], the GDP of G-10 economies is ranked among the top 23 high economies in the world. The GPD of Japan, Germany, United Kingdom, France, Italy, and Canada is ranked 3rd, 4th, 5th, 7th, 8th, and 9th, respectively, in the world. Several studies focused on the association between GDP and CO_2_e in different regions across the world producing different results [3,7,29,30,31,32]. For example, [9] found an increase in GDP and imports leads to an increase in CO_2_e in G-7 countries. On the other hand, [33] found that an increase in GDP leads to better environmental quality with the aid of advanced technologies and clean energy consumption. 

During the last two decades, industrialization and urbanization has brought harmful environmental changes [10,29,34,35,36,37,38]. Moreover, the demand of energy consumption is also increasing in developed countries, which in turn increases CO_2_e [10,39]. Progress has been made in every domain of technology, economic, and environmentally friendly policies [40]. All of these developments and advancements would not be possible without accounting for the effect on climate. Previous studies investigated the impact of institutional quality and renewable energy consumption (REC) on CO_2_e [9,41], but they ignored the effect of financial institutional deepening (FID; constructed from financial institutional depth, financial institutional access, and financial institutional efficiency) and technology innovations on CO_2_e. Therefore, this study adds to the existing literature on environmental quality and makes up for the shortfall in the existing body of knowledge in four ways. Firstly, this study investigates the impacts of financial institutional deepening on CO_2_e. Secondly, this study employs second generation methodology to panel data for G-10 countries. Thirdly, the study also includes other variables of GDP, import, export, renewable energy consumption, and technology innovations in G-10 countries from 1990 to 2020. Fourthly, this work applies the novel wavelet coherence approach to present the lead–lag relationship among the parameters. This method also explains the co-movement (positive/negative relationship) of the dependent and independent parameters.

The layout of the manuscript is as follows: the first section describes the introduction of the study, and the second section reviews the existing literature. The third section includes theoretical approaches, model building, datasets, and its sources. The fourth section includes empirical findings and discussions, and the fifth section offers conclusions and policy implications.

## 2. Literature Review

The relationship between CO_2_e, globalization, trade, gross domestic products (GDP), energy consumption per capita (EC), and institutional quality has attracted both the regulatory authorities and researchers worldwide [6,13,14,42,43,44,45,46,47,48,49,50]. Empirical results of earlier studies revealed either positive or negative relationships, and either supporting or contradicting results. For example, [51] investigated the relationship between clean EC, environmental sustainability, and economic growth by using the ARDL technique, and found that clean EC does not help reduce CO_2_e in the long run, whereas [52] found that clean EC positively affects economic output and negatively affects CO_2_e. They also found negative impacts of political globalization and CO_2_e across G-20, OECD, and EU countries. Tariq et al. [7] studied the existence of an environmental Phillips-curve between REC, GDP, unemployment, population, and non-renewable energy use in South Asian countries from 1991 to 2019. From the model, they found positive effects of GDP, non-renewable energy consumption, and population on environmental degradation, whereas REC and unemployment decreases environmental dilapidation. Wei et al. [53] found that there exists an Environmental Kuznets Curve (EKC) between natural resources abundance, globalization, GDP, ageing people, and CO_2_e for G-11 countries. Rahman et al. [54] studied consumption based CO_2_e in SAARC countries using the Fully Modified Ordinary Least Square and causality models from 1972 to 2015. They revealed that more than 62.39% of CO_2_e is from household consumption, with India being the highest contributor (37.27%). Hao [55] studied effect of economic growth, financial development index, industrialization and trade openness, REC, and human development on climate change in high, middle, and lower income countries from 1990 to 2020. They found that REC and trade openness helps in reducing CO_2_e in high and lower income countries. Majewska and Gierałtowska [56] studied the economic affluence on CO_2_e in Central and Eastern Europe from 2000 to 2019. They found that EC and REC are the keys factors which increase and decrease CO_2_e, respectively, in Central and Eastern Europe.

They also revealed the negative influences of political globalization and CO_2_e across G-20, OECD, and EU countries. Adebayo et al. [57] found both positive and negative fluctuations in GDP results in environmental degradation in Chile from 1990 to 2018. Both [58] and [51] found that enhanced economic growth leads to an increase in CO_2_e. Khan et al. [35] found a positive relationship between CO_2_e, import, and GDP, and a negative relationship between CO_2_e, REC, and export and technology innovations in G-7 countries during [59], which studied aggregate domestic consumption spending (ADCS) and technology innovations shocks on industrialization in South Africa from 1980 to 2014. They revealed that positive shocks in technology innovations and ADCS had positive effects on development in industrial sectors. In [60,61], the relationship between REC and technology innovations was investigated and it was found that use of renewables leads to an increase in air quality. 

Studies also focused on the association between institutional quality and CO_2_e [9,62,63,64], using wavelet coherence applications for environmental tasks [31,65,66,67,68,69]. To the best of the author’s knowledge, only a single study was conducted to evaluate the dynamic relationship between financial deepening and CO_2_e [12]. However, they overlooked the impact of financial institutional deepening on CO_2_e. Therefore, the present study investigates the relationship between CO_2_e, financial institutional deepening, GDP, import, export, and REC and technology innovations in G-10 countries from 1990 to 2020 using the CS-ARDL approach. The robustness of the CS-ARDL is confirmed by using the dynamic common correlated effects mean group (DCCEMG) model. Moreover, the novel wavelet coherence technique, which combines the time- and frequency-domain-based causality approach, is also applied to check the impact of magnitude of one parameter on another. The wavelet coherence approach allows us to carry out local analysis that captures the localized sub-image regions of a broader picture.

## 3. Theoretical Approach

Where *CO*_2_*e* and *FID* (financial institutional deepening) are the output and input series, respectively. While import, export, *REC*, technology innovations, and *GDP* are used as control variables to minimize excluded parameters bias in outcomes. So, we can write Equation (1) into panel data form as Equation (2):

(1) $$CO_{2\left(i,t\right)}e=f\left(IMP_{\left(i,t\right)},\;EXP_{\left(i,t\right)},\;GDP_{\left(i,t\right)}TI_{\left(i.t\right)},REC_{\left(i,t\right)},\;FID_{\left(i,t\right)}\right)$$

(2) $$CO_{2\left(i,t\right)}e=\Phi_{i,t}+\delta_{1}IMP_{i,t}+\delta_{2}EXP_{i,t}+\delta_{3}GDP_{i,t}+\delta_{4}TI_{i,t}+\delta_{5}REC_{i,t}+\delta_{6}FID_{i,t}+\mu_{i,t}$$

where $\delta_{1},\;\delta_{2},\;\delta_{3},\;\delta_{4\;,\;}\;\delta_{5},\;\;\mathrm{and}\;\delta_{6}$ represent coefficients of import, export, *GDP*, *REC* financial institutional deepening and technology innovations, respectively of country *i* in time *t*, whereas $\Phi_{i,t}\;\mathrm{and}\;\mu_{i,t}$ represent the constant and residual value, respectively. To minimize the consequences of heteroscedasticity, the entire data series in Equation (2) is log transformed [70,71]. Thus, the above equation becomes:

(3) $$lnCO_{2\left(i,t\right)}e=\Phi_{i,t}+\delta_{1}lnIMP_{i,t}+\delta_{2}lnEXP_{i,t}+\delta_{3}lnGDP_{i,t}+\delta_{4}lnTI_{i,t}+\delta_{5}lnREC_{i,t}+\delta_{6}lnFID_{i,t}+\mu_{i,t}$$

### 3.1. Model Building

Before checking the stationarity of the data, [72] cross-section dependence (CSD) and [73] tests are used to check the dependence in residual terms and heterogeneity in slope parameters, respectively. The Pesaran & Yamagata (2008) test is superior to [74] test and [75] test because it accounts for CSD. It is also applicable for a small sample size and longer period. Both of these tests are crucial as they help to identify the appropriate unit root test. Later, Cross-sectionally Augmented Dickey Fuller (CADF) and Im, Pesaran, and Shin CIPS unit root tests are used to check the data’s stationarity. Furthermore, the Durbin & Hausman test and the [76] co-integration test is used to investigate the relationship between CO_2_e, import, export, GDP, REC, TI, and financial institutional deepening. Finally, to investigate the long-run and short-run relationship amongst import, export, GDP, REC, TI, and financial institutional deepening, the best-known econometric approach to apply is the CS-ARDL model, designed and developed by [77]. The results obtained from the CS-ARDL approach are reliable irrespective of co-integration of the series [9]. Recently, the DCCEMG model, developed by Chudik et al. [78], is employed to limit the heterogeneity and endogeneity in slope. The residual CSD is used to determine the elastic effects of the explanatory parameters on the response parameter. Apart from cross-sectional averages (CSA), CS-ARDL by [77] is used to control cross-sectional correlations given as below:

(4) $$\begin{array}{l} lnCO_{2\left(i,t\right)}e=\Phi_{i}+\overset{p_{y}}{\underset{j=1}{\sum }}\lambda_{ij}lnCO_{2\left(it-j\right)}e+\overset{p_{x}}{\underset{j=0}{\sum }}\delta_{1j}lnIMP_{\left(it-j\right)}\\ \;\;\;\;\;\;+\overset{p_{x}}{\underset{j=0}{\sum }}\delta_{2j}lnEXP_{\left(it-j\right)}+\overset{p_{x}}{\underset{j=0}{\sum }}\delta_{3j}lnGDP_{\left(it-j\right)}\\ \;\;\;\;\;\;+\overset{p_{x}}{\underset{j=0}{\sum }}\delta_{4j}lnREC_{\left(it-j\right)}+\overset{p_{x}}{\underset{j=0}{\sum }}\delta_{5j}lnTI_{\left(it-j\right)}+\overset{p_{x}}{\underset{j=0}{\sum }}\delta_{6j}lnFID_{\left(it-j\right)}\\ \;\;\;\;\;\;+\overset{p}{\underset{j=0}{\sum }}\psi_{1j}{\bar{lnCO}}_{2\left(t-j\right)}e+\;\overset{p}{\underset{j=0}{\sum }}\psi_{2j}{\bar{lnIMP}}_{\left(t-j\right)}\\ \;\;\;\;\;\;+\overset{p}{\underset{j=0}{\sum }}\psi_{3j}{\bar{lnEXP}}_{\left(t-j\right)}+\overset{p}{\underset{j=0}{\sum }}\psi_{4j}{\bar{lnGDP}}_{\left(t-j\right)}\\ \;\;\;\;\;\;+\overset{p}{\underset{j=0}{\sum }}\psi_{5j}{\bar{lnREC}}_{\left(t-j\right)}+\overset{p}{\underset{j=0}{\sum }}\psi_{6j}{\bar{lnTI}}_{\left(t-j\right)}+\overset{p}{\underset{j=0}{\sum }}\psi_{7j}{\bar{lnFID}}_{\left(t-j\right)}\\ \;\;\;\;\;\;+\mu_{it} \end{array}$$

where ${\bar{lnCO}}_{2\;}e$, $\bar{lnIMP},\;\bar{lnEXP},\;{\bar{lnGDP}}_{},\;{\bar{lnREC}}_{},\;{\bar{lnTI}}_{}\;and\;{\bar{lnFID}}_{}$ represent the CSA of the response and the explanatory variables. $\Phi_{i}$ and $\lambda_{ij}$ show the impact specifications of unexamined countries and lagged co-efficients of the dependent parameter, respectively. $\delta_{1j}$, …, $\delta_{6j}$ and $\psi_{1j},\;\ldots ,\;\psi_{7j}$ are the variables of covariates and CSA of lagged series. The robustness of the CS-ARDL model is assessed by the DCCEMG approach. The DCCEMG model in line with the approach of Chudik & Pesaran, [77] and can be written as:

(5) $$\begin{array}{l} lnCO_{2\left(i,t\right)}e=\Phi_{i}+\lambda_{i}lnCO_{2\left(i,t-1\right)}e+\gamma_{1}lnIMP_{i,t}+\gamma_{2}lnEXP_{i,t}+\gamma_{3}lnGDP_{i,t}\\ \;\;\;\;\;\;+\gamma_{4}lnREC_{i,t}+\gamma_{5}lnTI_{i,t}+\;\gamma_{6}lnFID_{i,t}+\overset{K}{\underset{r=0}{\sum }}\Phi_{1ir}{\bar{lnCO}}_{2\left(i,t-1\right)}e\\ \;\;\;\;\;\;+\overset{K}{\underset{r=0}{\sum }}\Phi_{2ir}{\bar{lnIMP}}_{\left(i,t-1\right)}+\overset{K}{\underset{r=0}{\sum }}\Phi_{3ir}{\bar{lnEXP}}_{\left(i,t-1\right)}\\ \;\;\;\;\;\;+\;\overset{K}{\underset{r=0}{\sum }}\Phi_{4ir}{\bar{lnGDP}}_{\left(i,t-1\right)}+\overset{K}{\underset{r=0}{\sum }}\Phi_{5ir}{\bar{lnREC}}_{\left(i,t-1\right)}\\ \;\;\;\;\;\;+\overset{K}{\underset{r=0}{\sum }}\Phi_{6ir}{\bar{lnTI}}_{\left(i,t-1\right)}+\overset{K}{\underset{r=0}{\sum }}\Phi_{7ir}{\bar{lnFID}}_{\left(i,t-1\right)}+e_{it} \end{array}$$

Here, $\Phi_{1\mathrm{ir}}$, …, $\Phi_{5ir}$ and *K* represent the influence of independent variables on *CO*_2_*e* and mean lags, respectively. Lastly, Equation (6) depicts the [79] causality test employed to check causalities among the parameters as:

(6) $$Z_{it}=\delta_{i}+\overset{M}{\underset{m=1}{\sum }}\Phi_{i}^{\left(m\right)}Z_{it-m}+\overset{M}{\underset{m=1}{\sum }}\psi_{i}^{\left(m\right)}Y_{it-m}+e_{it}$$

where $\delta_{i}$, M, $\Phi_{i}^{\left(m\right)}$ and $\psi_{i}^{\left(m\right)}$ represent the fixed effects, order of lags, slope and coefficients of lag, respectively.

### 3.2. Datasets and Methodology

The panel datasets of CO_2_e, financial institutional deepening, technology innovations, GDP, REC, and import and export of G-10 countries during the period from 1990 to 2020 is used for analyses. The details of the datasets along with their source is given in Table 1. The missing data for some of the years limit our exploration to the aforesaid period. The summary statistics including mean, minimum, maximum, standard deviation (SD), skewness, kurtosis, and sample variance of all the parameters is portrayed in Table 2. The GDP has the highest mean value (1.68 × 10^12^ ± 1.22 × 10^12^), followed by technology innovations (4.40 × 10^5^ ± 6.10 × 10^3^), REC (12.91 ± 12.12), export (39.52 ± 19.51), import (36.74 ± 17.41), CO_2_e (0.75 ± 0.17), and financial institutional deepening (0.71 ± 0.09). The distribution of the series is positively skewed except those of CO_2_e and financial institutional deepening. When it comes to kurtosis, the distribution of technology innovations, import, and REC is leptokurtic in shape, whereas those of CO_2_e, technology innovations, GDP, and financial institutional deepening are platykurtic in shape. 

## 4. Empirical Findings and Discussion

The results acquired from several statistical approaches are discussed in this section. In the first step, we apply the [80] CSD test to check dependencies/independencies in the residuals. The results obtained from the [80] test (Table 3) reveal that null-hypothesis of no CSD amid the model’s residual terms were unacceptable. This signifies how the impact of one country will affect the others. The CSD test results of all variables are significant at 1% significant level. This revelation agrees with that of [81] for Western Africa but deviates from [82] for Northern China. Apart from this, disregarding the heterogeneity in the slope parameters could results in biased inferences [2,81].

Hence, following the research of [2,83], we used the [73] test to assess the homogeneity in slope parameters. The results of the [73] test in Table 4 reveal that there exists heterogeneity in slope parameters, indicating significant variations in the G-10 countries. The results portrayed in the Table 4 are significant at 5% and 10% significance level, respectively.

Afterwards, the cointegration analysis was performed by using the [84] test, the outcomes of which are portrayed in Table 5. The Gt and Ga signify the mean information of a group while Pt and Pa represent the overall panel information. The LR relationship is revealed from the estimated results at 1% significance level. The studies conducted by [9,35] for G-7 countries, [39] for NAFTA countries, [63] for South Asian countries, and [85] for G-20 countries support the above results. 

Furthermore, we apply the Cross-sectional Im, Pesaran, and Shin (CIPS) test and Cross-sectionally Augmented Dickey–Fuller (CADF) test to evaluate the stationarity properties of the parameters. The CIPS and CADF stationarity test results are given in Table 6. The outcomes of the tests show that the null hypothesis of non-stationarity for the entire series could not be rejected at levels but could at the first difference. This suggests that all the parameters acquired stationarity after the first difference, which portrays the homogeneous order of integration amongst the parameters. This order of integration describes the reason behind why the DCCEMG model is applied to analyze the long run association amid the whole series. Similar findings were offered by [9] for G-7 countries, [63] for three of the developing countries of Asia, [86] for South Asia, and [83] for North African countries.

### 4.1. CS-ARDL

The CS-ARDL approach is used to estimate the resilient effects of financial institutional deepening, technology innovations, GDP, REC, and import and export on CO_2_e in G-10 countries. The results presented in the Table 7 show that for every 1% increase in financial institutional deepening, environmental quality will deteriorate by 0.5403% and 0.2980% in the short run and long run, respectively, at a 1% significance level among G-10 countries. In [12], asymmetric consequences of financial institutional deepening on environmental quality in BRICS countries were studied and it was found that there were significant positive impacts of financial institutional deepening on CO_2_e in the long run. The results also reveal that imports positively impact CO_2_e, whereas exports negatively affect the CO_2_e in G-10 economies. Every 1% increase in import leads to an increase in CO_2_e by 0.2942% and 0.1479% in the short run and long run, respectively, whereas every 1% decrease in export will result in a decrease in CO_2_e by 0.3697% and 0.1906% in the short run and long run, respectively, amongst the G-10 countries. The effects of import and export on CO_2_e are significant because G-10 countries are the largest importers and exporters of carbon commodities in the world. Wahab et al. [87] found that exporting has an inverse relation with CO_2_e while importing has positive association with CO_2_e in G-7 countries. Khan et al. [35] observed that the elimination of tariffs among RCEP countries increases the global annual CO_2_e. Hasanov et al. [88] found statistically significant negative impacts of import and export on consumption-based CO_2_e. Moreover, our results for export contradict the findings of [89].

As far as REC is concerned, REC has negative impacts on CO_2_e. For instance, a 1% increase in REC lowers the CO_2_e by 0.0661% in the short run and 0.0354% in the long run at 10% significance level. The maximum REC was written down during the 1970s in Germany, 1990s in UK and 2000s in Japan, France, Canada, and Italy. In 2019, Sweden, Switzerland, France, Canada, Germany, Belgium, United Kingdom, Italy, Japan, and The Netherlands produced its 68.89%, 48.81%, 48.52%, 33.94%, 22.58%, 21.32%, 20.84%, 16.29%, 12.45%, and 7.53% of electricity, respectively, from low carbon emission sources, including wave and tidal, wind, solar, hydro-power, bioenergy, and geothermal energy. The topography of Sweden encourages the production of the highest percentage of energy from renewables worldwide. The carbon tax policy has been an outstanding way to address the problem of CO_2_e in Sweden. The continued renewable energy projects, including the partnership between the Swedish Government and Uniper Engineering and Fortum eNext for upraising hydrogen, wind and solar development, and hydro and physical trading optimization, reveals a devotion to and vigilance of environmental impacts, which are evident in the signing of the Paris Agreement by these countries. As of 1990, Germany remains successful in lowering CO_2_e by 40.8% by the end of 2020. This decline is associated with Germany’s climate policies, climate laws, and transition of the energy sector to renewable resources. Japan, after the United Kingdom and Germany, is the world’s third largest economy and has lowered 14% of GHG emissions between 2013 and 2019 through the use of innovative technology and renewable energy. Moreover, the American government has also promised to transform its heavy pollutant industries such as chemicals, aluminum, steel, concrete, and transport to lower carbon emissions through technology innovations and green procurement practices. 

In G-10 countries, negative coefficients of GDP per capita show the decarbonizing effects in high economies both in the long run (0.1444%) and short run (0.0514%). According to a report issued by the International Energy Agency and World Resources Institute, CO_2_e are decoupled, whereas countries’ GDP, including G-10 countries, is still growing [90]. Cai et al. [91] found that the GDP of developed countries does not influence CO_2_e, which might be due to their active policies and measures on degradation of environmental quality and climate change. Recently, the G-20 summit held in Japan in June 2019 urged other nations to contribute to net zero carbon emissions by 2050. Similar results have been found by [92] for United Kingdom, and [87] and [91] for G-7 countries, whereas contradicting results were obtained by [93]. Salari et al. [31] found a negative relationship between REC and CO_2_e. They also found an inverted U-shape relationship between CO_2_e and GDP. Technology innovations have a negative impact on CO_2_e both in the short run and in the long run. Furthermore, for every 1% rise in technology innovations, CO_2_e reduces by 0.0957% in short run and 0.0569% in the long run, respectively, among the G-10 nations. Wahab et al. [87] found an inverse relationship between technology innovations and energy resources producing CO_2_e for G-7 countries. They also suggest that promoting technology innovations and green, clean energy production reduces CO_2_e. Erdoğan et al. [85] studied the effects of technology innovations on energy, transport, and other industries. They found that an increase in technology innovations in industries leads to lowering of CO_2_e.

The DCCEMG model results portrayed in Table 8 illustrate the robustness of the CS-ARDL approach. The estimated values of the variables vary in significance and weights, but the similar signs of the two models indicate the vigorousness of the model. Moreover, similar trends in post-estimated statistics put further emphasis on the efficacy and credibility of this research. 

The results obtained from the DH causality test are portrayed in Table 9. The outcomes depict a bi-directional association between CO_2_e, GDP, financial institutional deepening, and import and export at 1% significance level. These outcomes suggest that appropriate environmental policies, regulation of pollutant capacities, and limiting imports of carbon commodities, can reduce CO_2_e in G-10 countries, whereas one-way causality exists from technology innovations to CO_2_e and CO_2_e to REC. This implies that strategic planning about the aforementioned factors helps to lower environmental pollution. 

### 4.2. Wavelet Coherence

**Figure 1:**
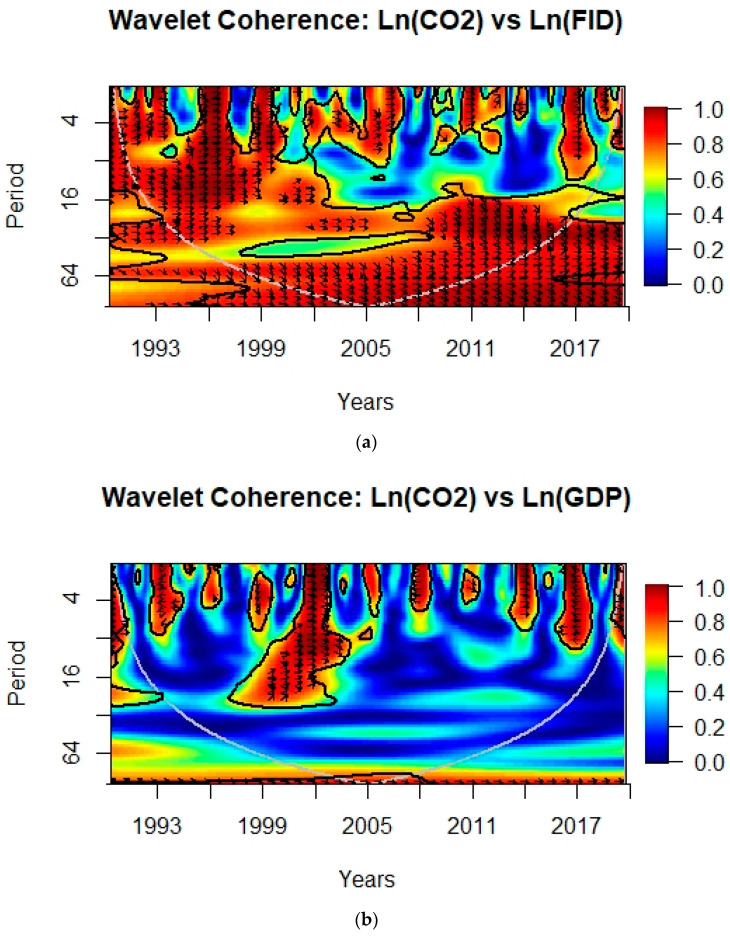

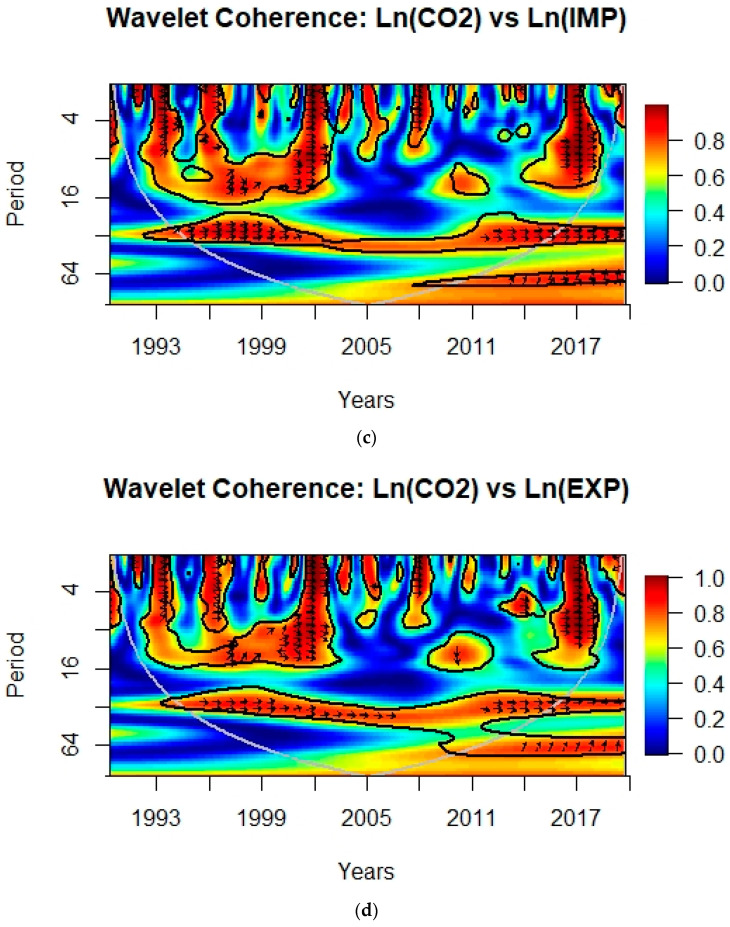

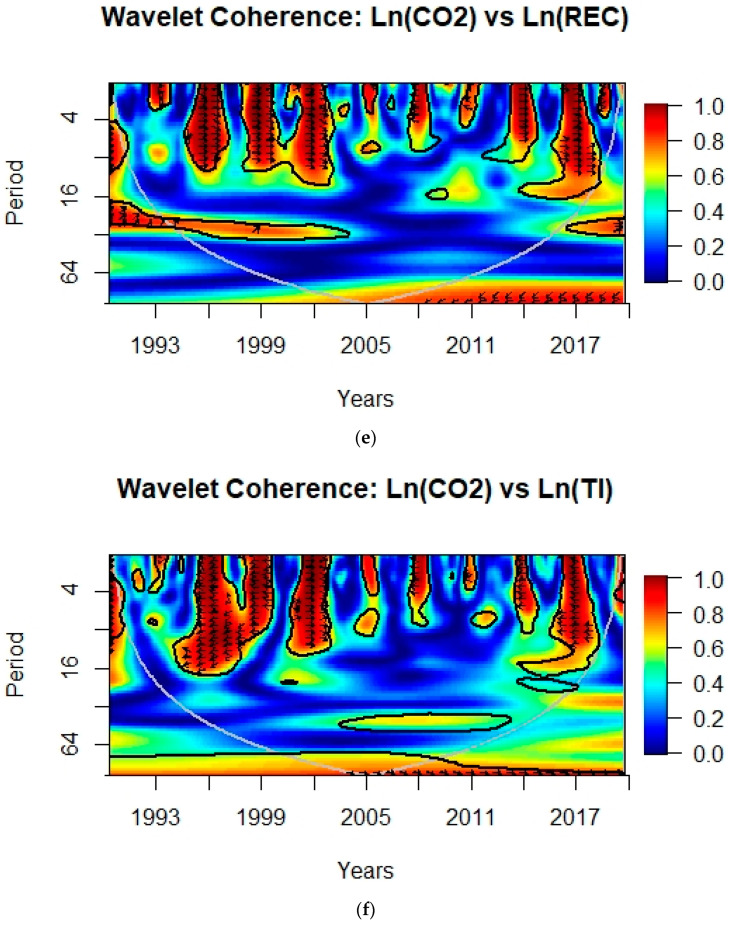
Shows the wavelet coherence between CO_2_e and (**a**) financial institutional deepening (FID), (**b**) GDP, (**c**) import (IMP), (**d**) export (EXP), (**e**) REC, and (**f**) technology innovation (TI) for G-10 countries from 1990 to 2020.

The wavelet coherence plots between CO_2_e and financial institutional deepening, GDP, import, export, REC, and Technology Innovation for G-10 countries from 1990 to 2020 has been shown in Figure 1. The outcomes of the wavelet coherence plot help to determine the correlation between the two parameters in a time–frequency space [64]. The x-axis shows the time in years from 1990 to 2020 and the y-axis shows the time period in the number of years or frequency or scale. The color bar shows the coherence from dark blue (lowest) to dark red (highest). The areas surrounded by black lines designate a 5% significance level related to the null hypothesis of a power spectrum, calculated by Monte Carlo simulations. The cone of influence, the area from the cone outline to the axes, shows areas that may be affected by edge effects (i.e., consequences appearing from wavelets overextended outside the limits of the monitored duration); the detected depiction of the facts in this area should be analyzed with carefulness [94]. The arrows in the right and left directions show the positive and negative significant relationships, respectively, between the two parameters, and arrows upward and downward show the lag–lead relationships among the variables (e.g., uncertainty) [95]. The rightward-up and leftward-down directions display a positive correlation with the second parameter leading (the second variable causes the first variable), whereas the rightward-down and leftward-up directions demonstrate a negative correlation with the first variable leading [96]. 

Figure 1a shows a high correlation (R^2^ = 0.9–1) of CO_2_e with financial institutional deepening at the period of scale 4–16 from 1993 to 1999. The arrows are in-phase indicating direct relationship between CO_2_e and financial institutional deepening from 1993 to 1997, which means that an increase in financial institutional deepening led to increase in CO_2_e in G-10 countries, whereas from 2009 to 2015, in the frequency band of 16–32, the rightward-down arrows show CO_2_e is leading the financial institutional deepening. Figure 1b, from 1999 to 2004 and 2013 to 2015, with time-period scales 0–28 and 0–8, respectively, show a high positive correlation between CO_2_e and GDP. A negative correlation between CO_2_e and GDP is observed for the 0–11 and 0–8 quarterly period from 1992 to 1994 and 2016 to 2018, respectively. Khalfaoui et al. [67] found bidirectional causality between CO_2_e and GDP using wavelet coherence scales in G-7 countries.

In Figure 1c, between the 0–16 period scale, from the years 1993 to1994, 2001 to 2003, and 2016 to 2018, a strong correlation with the arrows pointing to the right mostly are exhibited, indicating that the CO_2_ emissions are in-phase with import. From 1995 to 2001 and 2011 to 2015, a strong positive correlation between CO_2_e and import was also exhibited, around 32 periods of quarterly time scales. A strong positive in-phase correlation is observed between CO_2_e and export in the years 1992 to 1994, 1995 to 1997, 2001 to 2003, 2007 to 2009 and 2016 to 2018 between 0–16 periods of quarterly scales, and at the quarterly period of 28–48 in the years 1995 to 2006 and 2012 to 2015. Mutascu et al. [97] studied the relationship between CO_2_e and trade openness in the European Union using the wavelet coherence approach and found that CO_2_e was positively related to imports and exports from 2006 to 2010 and 2007 to 2008, respectively. 

The CO_2_e and REC wavelet coherence plot shows positive correlation regions during a 0–8 year time period from 1995 to 1997, 1998 to 2003, 2013 to 2015, and 2016 to 2018. There was a negative relationship between CO_2_e and REC from 2007 to 2008 and 2010 to 2011, on a 0–4 scale. Alola and Kirikkaleli [65] found a positive correlation between CO_2_e and renewable consumption in the USA. The wavelet coherence plot between CO_2_e and technology innovations shows high negative correlations for the years 1994 to 2000 and 2016 to 2018, on a 0–16 quarterly scale of period: from 2008 to 2009 and 2013 to 2015 on a 0–6 scale in the short run. The direct relationship was from 2001 to 2003 for a quarterly period of 0–12. Adebayo et al. [96] found negative relationships between CO_2_e and technology innovations, and a positive relationship between CO_2_e and GDP, with GDP leading the CO_2_e.

## 5. Conclusions

In this study, we examined the association among CO_2_e, GDP, import, export, REC, technology innovations, and financial institutional deepening in G-10 countries using the CS-ARDL approach from 1990 to 2020. The effectiveness and reliability of the CS-ARDL technique is confirmed by using the DCCEMG model. We also employed CSD, CIPS and CADF unit root tests, slope heterogeneity test, and Dumitrescu & Hurlin (2012) causality tests in the analysis. For the cointegration relationship, we used the Westerlund [86] test to check the heterogeneity in slope that confirms the cointegration relationship amid the time–series parameters.

The results of CS-ARDL show that financial institutional deepening and imports positively impact CO_2_e in G-10 economies. For instance, a 1% increase in financial institutional deepening and import will increase CO_2_e by 0.5403% and 0.2942% in the short run and 0.2980% and 0.1479% in the long run, respectively, at a 1% significance level. On the other hand, GDP, export, technology innovations, and REC negatively affect the CO_2_e in G-10 countries. For every 1% increase in GDP, export, technology innovations, and REC will result in a decrease in CO_2_e by 0.1444%, 0.3697%, 0.0957%, and 0.0661% for the short run, and 0.0514%, 0.1906%, 0.0569%, and 0.0354% in the long run, respectively.

A high correlation (R^2^ = 0.9–1) is found between CO_2_e and financial institutional deepening at higher frequencies from 1993 to 1999, whereas, from 2009 to 2015, the rightward-down arrows show that CO_2_e is leading the financial institutional deepening in the medium frequency band of 16–32. A negative correlation between CO_2_e and GDP is observed for the 0–11 frequency band from 2016 to 2018 for G-10 economies. A strong positive correlation is present in the 8–16 year period between CO_2_e and import. The CO_2_e cycled upward with export, revealing that CO_2_e led export by π × 2^−1^ at multiple scales. The CO_2_e and REC wavelet coherence plot shows a distinct higher frequency and correlation regions during the 1–8 year time period. The CO_2_e and technology innovations were out of phase from 1994 to 2000, 2008, 2014, and 2017, respectively, depicting and inverse relationship between them and were in-phase from 2002 to 2003, indicating a direct relationship between them. 

## 6. Policy Recommendations

This work shows that these countries need to consider import and financial institutional deepening to lower their environmental pollution. In this regard, it is important to consider the financial assets of the G-10 nations. Therefore, these countries need to consider the financial markets and firms to revise the current environmental policies. In doing so, G-10 countries can provide financial incentives to the firms and markets to make their workings environmentally friendly. These countries should also lower the import of carbon commodities, which in turn reduces CO_2_e. Our findings endorse the role of renewable energy in improving air quality in G-10 nations. The increase in renewable energy consumption, use of energy efficient technologies, and suitable environmental policies for financial institutional deepening and import will assist the G-10 economies in environmental sustainability. The exports and economic growth also contribute towards environmental sustainability. The DH causality test further confirms that renewable energy consumption, import, export, GDP, and technology innovations significantly impact CO_2_e in G-10 countries.

Apart from the contribution, this work has some limitations that can be filled by future research work. Future works can analyze the time series data for other group of countries by including other socio-economic factors.

## Figures and Tables

**Table 1:** Represents the parameters under investigation and their sources.

Parameters	Abbreviation	Measurement	Source
Carbon-Dioxide Emissions	CO_2_e	Metric ton	World Bank
Financial Institutional Deepening	FID	FID is constructed from financial institutional depth, access, and efficiency	IMF
Technology Innovations	TI	Patent applications, residents, and non-residents	World Bank
Gross Domestic Products	GDP	constant 2015 US$	World Bank
Renewable Energy Consumption	REC	Percentage of total final energy consumption	World Bank
Import	IMP	Percent of GDP	World Bank
Export	EXP	Percent of GDP	World Bank

**Table 2:** Represents the descriptive statistics of the parameters.

Parameters	Mean	Min	Max	SD	Skewness	Kurtosis	Sample Variance
CO_2_e	0.75	0.27	1.0	0.17	−0.25	−0.60	0.03
FID	0.71	0.48	0.88	0.09	−0.45	−0.22	0.01
TI	5.21 × 10^4^	617	4.40 × 10^5^	6.10 × 10^3^	2.62	5.31	1.16 × 10^10^
GDP	1.68 × 10^12^	2.83 × 10^11^	4.59 × 10^12^	1.22 × 10^12^	0.67	−0.61	1.48 × 10^24^
REC	12.91	0.61	52.89	12.12	1.45	1.86	146.83
IMP	36.74	6.94	83.28	17.41	0.79	0.06	303.01
EXP	39.52	8.82	84.68	19.51	0.67	−0.52	380.63

**Table 3:** Results obtained for CSD analysis.

Variable	Test Statistics (*p*-Values)
CO_2_e	29.99 *** (0.00)
FID	35.84 *** (0.00)
TI	27.78 *** (0.00)
GDP	37.35 *** (0.00)
REC	33.59 *** (0.00)
IMP	37.29 *** (0.00)
EXP	37.30 *** (0.00)

*** is significant at 1%.

**Table 4:** Results of Slope heterogeneity test.

Statistics	Test Value (*p*-Value)
Delta tilde	−1.79 * (0.073)
Delta tilde Adjusted	−2.16 ** (0.031)

** = 5% and * = 10%.

**Table 5:** Cointegration test.

Statistic	Values
Gt	−2.206
Ga	−10.272 ***
Pt	−5.226
Pa	−7.619 ***

*** is significant at 1%.

**Table 6:** CIPS and CADF unit root test.

Variable		CIPS Test	CADF Test	
	At Level	First Difference	At Level	First Difference
**CO_2_e**	−2.43	−4.78 ***	−2.17	−2.83 **
**FID**	−3.39	−5.94 ***	−2.75	−3.94 ***
**TI**	−3.90	−6.45 ***	−2.78	−4.00 ***
**GDP**	−2.55	−3.89 **	−2.42	−3.18 ***
**REC**	−3.61	−6.13 ***	−2.78	−4.08 ***
**IMP**	−2.52	−5.03 ***	−3.18	−4.21 ***
**EXP**	−1.94	−4.07 ***	−2.95	−3.43 ***

*** is significant at 1% and ** at 5%.

**Table 7:** CS-ARDL.

	Dependent Variable: CO_2_e					
Variable	Short Run			Long Run		
	Coefficients	Std. Error	Significance	Coefficients	Std. Error	Significance
ΔlnFID	0.5403 ***	0.1210	0.000	0.2980 ***	0.0682	0.000
ΔlnTI	−0.0957 **	0.0966	0.022	−0.0569 *	0.0539	0.092
ΔlnGDP	−0.1444 **	0.2983	0.028	−0.0514 **	0.1537	0.038
ΔlnREC	−0.0661 *	0.0715	0.053	−0.0354 *	0.0389	0.062
ΔlnIMP	0.2942 **	0.1448	0.042	0.1479 ***	0.0717	0.007
ΔlnEXP	−0.3697 ***	0.1340	0.006	−0.1906 **	0.0708	0.039

*** = significant at 1%, ** = significant at 5%, and * = significant at 10%.

**Table 8:** Shows results obtained from DCCEMG model.

Dependent Parameter: CO_2_e		DCCEMG	
	Coefficient	Std. Error	Significance
FID	0.7435 ***	0.1566	0.000
TI	−0.0560 *	0.0745	0.052
GDP	−0.8008 **	0.2581	0.002
REC	−0.0593 *	0.1012	0.058
IMP	0.0790 **	0.1581	0.017
EXP	−0.0697 **	0.1260	0.080

*** = significant at 1%, ** = significant at 5%, and * = significant at 10%.

**Table 9:** Pairwise Dumitrescu & Hurlin, (2012) panel causality test results.

Null Hypothesis	W-Stat.	Z-Bar-Stat.	Prob.
lnFID ⇏ lnCO_2_	5.0161 ***	3.7501	0.000
lnCO_2_ ⇏ lnFID	5.7351 ***	4.7013	0.000
lnGDP ⇏ lnCO_2_e	4.7603 ***	3.4115	0.001
lnCO_2_e ⇏ lnGDP	6.3406 ***	5.5025	0.000
lnTI ⇏ lnCO_2_e	5.6951 ***	4.6434	0.000
lnCO_2_e ⇏ lnTI	3.5401 *	1.7944	0.073
lnREC⇏ lnCO_2_e	2.6809 *	0.6604	0.509
lnCO_2_e ⇏ lnREC	4.8328 ***	3.5076	0.000
lnCO_2_e ⇏ lnEXP	9.7368 ***	9.9959	0.000
lnEXP ⇏ lnCO_2_e	4.9428 ***	3.6531	0.000
lnIMP ⇏ lnCO_2_	4.9739 ***	3.6943	0.000
lnCO_2_ ⇏ lnIMP	10.9025 ***	11.5383	0.000

1% = ***, 10% = *.

## Data Availability

Data used and their sources are given in the manuscript.
